# Supplementary material for: A Likelihood Approach for Real-Time Calibration of Stochastic Compartmental Epidemic Models
Source: PLoS Comput Biol. 2017 Jan 17;13(1):e1005257. doi: 10.1371/journal.pcbi.1005257 (PMC5240920; doi:10.1371/journal.pcbi.1005257)
Supplement: S1 File — (TAR.GZ) [file pcbi.1005257.s014.tar.gz › HSPH_Online-SI-Revision/EnKF07/n10-mild/n10-mild_EnKF_table-8.pdf]

| | | | | | | | | | | | | | | | | | | | | | | | | | | | |
|---|---|---|---|---|---|---|---|---|---|---|---|---|---|---|---|---|---|---|---|---|---|---|---|---|---|---|---|
| 1 | R0 | {1.25046, 6.80278} | {2.11412, 9.24801} | {{0.49769, 6.24872}, {3., 14.5922}} | {102.477, 50.1198} | inf | 59. | 524.714 | {321.122, 999.066} | 791.859 | 0.823371 | newly | 76. | 396.23 | {118., 825.} | 425.882 | 0.541761 | Reff | 1.22245 | 0.396754 | {0.0568947, 0.670902} | 67.7901 | ar | 3516. | 18 069.6 | {16 900., 22 394.} | 413.926 |
| 2 | R0 | {1.32013, 4.45847} | {2.40275, 5.77604} | {{1.75996, 1.29526}, {3., 13.1904}} | {84.8786, 78.7471} | inf | 43. | 2476.7 | {501.214, 5480.91} | 5660.29 | 1.57137 | newly | 76. | 2619.4 | {477., 7241.} | 3347.5 | 1.33913 | Reff | 1.30046 | 0.666545 | {0.214825, 1.34556} | 52.8809 | ar | 4420. | 15 544.4 | {7565., 25 681.} | 253.113 |
| 3 | R0 | {1.31785, 5.36796} | {2.27939, 10.8101} | {{0.890369, 2.02675}, {3., 16.9861}} | {87.2435, 116.426} | inf | 49. | 2457.24 | {547.728, 4772.57} | 4916.24 | 1.55851 | newly | 66. | 2209.21 | {418., 3978.} | 3249.68 | 1.32728 | Reff | 1.29953 | 1.29148 | {0.211038, 2.21995} | 45.7486 | ar | 4942. | 13 045.3 | {8867., 17 434.} | 163.968 |
| 4 | R0 | {1.38801, 6.16031} | {1.9989, 4.26758} | {{0.166654, 0.863691}, {3., 6.00662}} | {89.6716, 43.5203} | inf | 59. | 470.893 | {152.204, 753.604} | 699.36 | 0.774097 | newly | 85. | 515.14 | {118., 1032.} | 509.035 | 0.614364 | Reff | 1.35636 | 0.264359 | {0.0248597, 0.461489} | 80.5097 | ar | 4790. | 16 506.7 | {15 370., 18 085.} | 245.401 |
| 5 | R0 | {1.27955, 11.57} | {2.04941, 5.77504} | {{0.051095, 1.88706}, {3., 9.97674}} | {117.346, 52.2003} | inf | 102. | 313.709 | {75.4282, 300.93} | 218.803 | 0.251771 | newly | 64. | 417.05 | {62., 237.} | 561.203 | 0.384633 | Reff | 1.19011 | 0.394604 | {0.00706329, 1.30261} | 82.7676 | ar | 3460. | 17 756.2 | {16 268., 21 260.} | 413.185 |
| 6 | R0 | {1.22184, 4.34395} | {2.21089, 9.32489} | {{0.593134, 2.10795}, {3., 12.8451}} | {102.221, 134.312} | inf | 20. | 1842.36 | {311.01, 4126.47} | 9113.77 | 1.81737 | newly | 28. | 2243.7 | {328., 7120.} | 7916.14 | 1.66883 | Reff | 1.21683 | 1.52431 | {0.257156, 2.52477} | 60.7006 | ar | 3524. | 11 695.5 | {5008., 19 540.} | 232.652 |
| 7 | R0 | {1.23856, 4.59068} | {2.40666, 5.0728} | {{1.7811, 0.841749}, {3., 13.2084}} | {97.118, 73.3639} | inf | 52. | 2455.99 | {493.77, 5485.81} | 4624.1 | 1.50004 | newly | 78. | 2823.19 | {442., 7986.} | 3520.58 | 1.32519 | Reff | 1.20995 | 0.563142 | {0.156128, 1.34297} | 58.2404 | ar | 3855. | 14 788.6 | {7193., 25 684.} | 284.643 |
| 8 | R0 | {1.34531, 10.6689} | {2.10633, 9.32099} | {{0.487584, 5.97414}, {3., 14.5328}} | {93.1697, 40.6914} | inf | 98. | 528.739 | {310.376, 868.323} | 450.103 | 0.658254 | newly | 72. | 406.71 | {114., 744.} | 475.986 | 0.640943 | Reff | 1.29352 | 0.403765 | {0.0443991, 0.667661} | 69.3552 | ar | 4852. | 18 149.8 | {16 930., 20 390.} | 274.069 |
| 9 | R0 | {1.31775, 5.19226} | {2.17711, 4.2707} | {{0.992918, 2.67827}, {3., 5.12616}} | {78.1653, 23.6863} | inf | 51. | 1603.8 | {913.927, 5244.12} | 3044.7 | 1.35205 | newly | 85. | 1651.82 | {681., 5376.} | 1845.74 | 1.13437 | Reff | 1.29522 | 0.318317 | {0.0515226, 0.48571} | 75.4236 | ar | 4708. | 15 493.5 | {6961., 26 106.} | 232.387 |
| 10 | R0 | {1.25916, 6.89207} | {2.34907, 5.80723} | {{1.47295, 2.41883}, {3., 10.0026}} | {86.5582, 55.4142} | inf | 121. | 289.64 | {28.4752, 709.947} | 156.2 | 0.373077 | newly | 119. | 541.5 | {83., 1318.} | 377.479 | 0.456659 | Reff | 1.17404 | 0.633325 | {0.241067, 1.47532} | 55.7834 | ar | 3823. | 18 577.3 | {17 052., 24 296.} | 385.934 |
| 11 | R0 | {1.20377, 5.46566} | {2.48521, 7.12049} | {{2.12603, 3.82557}, {3., 14.7181}} | {106.972, 49.8989} | inf | 61. | 2043.6 | {485.132, 4432.56} | 3253.63 | 1.37686 | newly | 77. | 2267.62 | {323., 5788.} | 2848. | 1.26965 | Reff | 1.17392 | 0.932236 | {0.343305, 2.04155} | 47.992 | ar | 3114. | 14 586.6 | {8939., 21 159.} | 368.419 |
| 12 | R0 | {1.27878, 2.55804} | {2.24532, 10.9763} | {{0.886393, 4.28924}, {3., 16.9542}} | {87.6346, 333.117} | inf | 6. | 2601.16 | {361.618, 5326.1} | 43 253.7 | 2.41563 | newly | 12. | 2484.97 | {163., 5236.} | 20 611.3 | 2.02049 | Reff | 1.27328 | 1.37739 | {0.30187, 2.21915} | 44.4721 | ar | 3930. | 12 815.8 | {6231., 17 667.} | 226.403 |
| 13 | R0 | {1.41227, 5.45696} | {2.23439, 9.06489} | {{0.496976, 5.93489}, {3., 14.5648}} | {85.3539, 66.1611} | inf | 41. | 511.417 | {314.734, 743.541} | 1155.5 | 0.975837 | newly | 62. | 368.56 | {117., 715.} | 505.194 | 0.697487 | Reff | 1.38233 | 0.378622 | {0.098197, 0.569005} | 72.6098 | ar | 4822. | 18 147.8 | {16 954., 20 282.} | 276.353 |
| 14 | R0 | {1.32917, 7.27593} | {2.30765, 5.24965} | {{1.4902, 2.48172}, {3., 10.0958}} | {73.6161, 55.1012} | inf | 80. | 400.364 | {174.361, 798.242} | 403.471 | 0.486964 | newly | 96. | 748.58 | {94., 3015.} | 683.229 | 0.513507 | Reff | 1.28969 | 0.642158 | {0.238821, 1.47684} | 59.0377 | ar | 4758. | 18 491.9 | {16 932., 21 700.} | 288.649 |
| 15 | R0 | {1.31629, 9.97481} | {1.96264, 4.68962} | {{0.162183, 0.887693}, {3., 5.96463}} | {96.2204, 58.8758} | inf | 84. | 410.406 | {100.225, 642.824} | 391.948 | 0.580633 | newly | 70. | 433.5 | {86., 729.} | 523.857 | 0.6363 | Reff | 1.2843 | 0.268309 | {0.0247302, 0.461096} | 79.1086 | ar | 4543. | 16 278.2 | {15 404., 17 925.} | 259.484 |
| 16 | R0 | {1.36735, 5.74166} | {2.13617, 4.56165} | {{1.19227, 2.67893}, {3., 5.16732}} | {67.3567, 31.8214} | inf | 40. | 1595.89 | {580.271, 5431.6} | 3889.73 | 1.42512 | newly | 65. | 1719.63 | {377., 5439.} | 2547.52 | 1.22195 | Reff | 1.34356 | 0.382508 | {0.0369148, 0.792181} | 75.0815 | ar | 4516. | 16 472.8 | {9739., 25 115.} | 265.552 |
| 17 | R0 | {1.27524, 13.2396} | {2.39655, 6.30063} | {{1.49541, 2.4761}, {3., 10.1162}} | {87.9294, 52.4107} | inf | 39. | 298.239 | {168.628, 487.077} | 673.672 | 0.758802 | newly | 23. | 606.23 | {85., 2257.} | 2545. | 1.0472 | Reff | 1.26504 | 0.58768 | {0.0300625, 1.49527} | 63.1929 | ar | 3731. | 18 137. | {16 773., 23 080.} | 386.115 |
| 18 | R0 | {1.21884, 7.71545} | {2.27036, 5.01639} | {{1.48666, 2.44342}, {3., 10.0511}} | {86.272, 54.4111} | inf | 68. | 322.476 | {160.371, 680.176} | 383.897 | 0.552148 | newly | 61. | 677.96 | {88., 2347.} | 1017.44 | 0.694456 | Reff | 1.17923 | 0.645442 | {0.237145, 1.49883} | 57.7541 | ar | 3080. | 18 480.3 | {16 937., 23 185.} | 500.008 |
| 19 | R0 | {1.34879, 6.71464} | {2.12619, 4.48787} | {{1.16464, 2.67994}, {3., 5.14809}} | {72.3609, 39.2255} | inf | 96. | 1495.35 | {414.449, 5406.85} | 1458.33 | 1.05767 | newly | 111. | 1709.11 | {604., 5470.} | 1440.91 | 1.01539 | Reff | 1.29578 | 0.42225 | {0.0897213, 0.927376} | 71.2379 | ar | 4585. | 16 095.1 | {7318., 26 304.} | 251.185 |
| 20 | R0 | {1.37284, 5.75036} | {2.39977, 5.12286} | {{1.67004, 0.773754}, {3., 13.1167}} | {77.7405, 65.3523} | inf | 46. | 2342.13 | {495.658, 5485.25} | 4991.59 | 1.53264 | newly | 83. | 2670.62 | {479., 7287.} | 3117.61 | 1.30728 | Reff | 1.34758 | 0.638063 | {0.254648, 1.34443} | 54.4884 | ar | 4750. | 15 407.7 | {9858., 25 324.} | 225.973 |
| 21 | R0 | {1.29938, 5.36419} | {2.3966, 4.77877} | {{1.6764, 0.808644}, {3., 12.7823}} | {87.277, 62.5904} | inf | 36. | 2329.49 | {501.321, 5480.21} | 6370.8 | 1.64771 | newly | 59. | 2678.92 | {496., 7527.} | 4440.54 | 1.47382 | Reff | 1.28145 | 0.615018 | {0.245144, 1.26456} | 54.5146 | ar | 4337. | 15 312.8 | {7539., 25 431.} | 255.182 |
| 22 | R0 | {1.31882, 7.47626} | {2.23585, 5.21083} | {{1.48727, 2.49532}, {3., 10.1291}} | {69.534, 54.5614} | inf | 67. | 415.267 | {172.055, 1015.32} | 526.338 | 0.588438 | newly | 68. | 821.22 | {92., 4006.} | 1111.68 | 0.666547 | Reff | 1.28479 | 0.673348 | {0.0398577, 1.50011} | 59.1633 | ar | 3977. | 18 701.9 | {16 779., 24 980.} | 370.251 |
| 23 | R0 | {1.26215, 3.46375} | {2.61057, 7.45103} | {{2.13253, 4.16929}, {3., 14.6745}} | {107.696, 115.115} | inf | 5. | 2014.82 | {642.631, 3253.6} | 40 196.3 | 2.46698 | newly | 32. | 2071.35 | {608., 5162.} | 6374.84 | 1.6351 | Reff | 1.25672 | 0.89345 | {0.315599, 2.05554} | 51.1155 | ar | 3607. | 14 700.5 | {10 422., 21 409.} | 307.78 |
| 24 | R0 | {1.16712, 4.19464} | {2.54728, 5.45877} | {{1.18164, 4.04434}, {3., 7.64754}} | {119.387, 48.1964} | inf | 63. | 199.609 | {40.4175, 303.772} | 230.535 | 0.443348 | newly | 109. | 219.48 | {67., 486.} | 129.193 | 0.32662 | Reff | 1.13853 | 0.568256 | {0.164724, 1.36788} | 64.7188 | ar | 3000. | 17 926.3 | {16 490., 21 690.} | 497.543 |
| 25 | R0 | {1.22751, 3.34517} | {2.2118, 9.0987} | {{0.591001, 2.10806}, {3., 15.2272}} | {98.3328, 190.1} | inf | 2. | 1875.94 | {574.539, 3721.21} | 93 697.2 | 2.84481 | newly | 9. | 2549.23 | {415., 7941.} | 28 224.8 | 2.21413 | Reff | 1.22628 | 1.50895 | {0.250186, 2.11545} | 58.3239 | ar | 3086. | 11 702.5 | {5333., 18 443.} | 279.266 |
| 26 | R0 | {1.18747, 5.88083} | {2.15638, 9.21564} | {{0.470858, 5.82559}, {3., 14.462}} | {113.669, 58.1256} | inf | 134. | 518.843 | {33.1866, 756.949} | 306.649 | 0.517417 | newly | 150. | 398.59 | {14., 615.} | 195.78 | 0.414162 | Reff | 1.11017 | 0.384698 | {0.0569773, 0.672541} | 65.6058 | ar | 3614. | 18 113.3 | {16 896., 20 298.} | 401.197 |
| 27 | R0 | {1.29023, 4.76202} | {2.4646, 5.60731} | {{2.0249, 4.04336}, {3., 7.81922}} | {92.3617, 35.636} | inf | 32. | 187.735 | {7.3207, 257.922} | 506.548 | 0.714408 | newly | 65. | 236.13 | {6., 465.} | 287.862 | 0.567055 | Reff | 1.26391 | 0.635937 | {0.187793, 1.86732} | 65.7466 | ar | 4245. | 18 122.3 | {16 453., 24 680.} | 328.619 |
| 28 | R0 | {1.2258, 12.9056} | {2.86816, 6.27108} | {{2.49293, 2.22161}, {3., 15.641}} | {133.983, 62.0443} | inf | 157. | 218.977 | {50.0394, 532.495} | 88.402 | 0.290417 | newly | 86. | 472.98 | {20., 676.} | 473.279 | 0.591885 | Reff | 1.08814 | 0.988223 | {0.502084, 2.38203} | 55.8133 | ar | 3639. | 15 220.8 | {12 932., 21 633.} | 318.269 |
| 29 | R0 | {1.34702, 6.29049} | {1.96199, 4.61362} | {{0.160251, 0.891343}, {3., 5.96706}} | {92.9726, 41.7562} | inf | 78. | 421.096 | {97.6583, 650.734} | 443.135 | 0.614446 | newly | 109. | 440.34 | {90., 818.} | 314.44 | 0.488707 | Reff | 1.31765 | 0.266401 | {0.0245867, 0.46072} | 79.7822 | ar | 4560. | 16 411.4 | {15 420., 17 930.} | 260.386 |
| 30 | R0 | {1.37931, 6.78741} | {2.47466, 5.71071} | {{1.28093, 4.22222}, {3., 7.8546}} | {83.2599, 28.453} | inf | 121. | 258.202 | {17.3858, 269.455} | 146.932 | 0.315904 | newly | 153. | 265.62 | {11., 501.} | 134.288 | 0.429641 | Reff | 1.31324 | 0.498815 | {0.181872, 1.05144} | 68.0346 | ar | 4667. | 17 868. | {16 670., 19 326.} | 282.858 |
| 31 | R0 | {1.16062, 4.47529} | {2.21078, 9.32619} | {{0.592693, 2.10806}, {3., 12.8432}} | {110.987, 128.606} | inf | 8. | 1854.7 | {333.562, 4126.43} | 23 083.7 | 2.2134 | newly | 12. | 2245.54 | {388., 6971.} | 18 612.8 | 2.01803 | Reff | 1.1576 | 1.5246 | {0.257068, 2.52433} | 65.8395 | ar | 3037. | 11 712.8 | {5055., 19 551.} | 285.803 |
| 32 | R0 | {1.37647, 18.7122} | {2.75434, 2.58094} | {{2.2831, 1.92047}, {3., 4.64123}} | {100.102, 86.2072} | inf | 284. | 206.149 | {42.3778, 204.731} | 107.825 | 0.643702 | newly | 111. | 646.25 | {69., 1581.} | 518.405 | 0.414542 | Reff | 1.17055 | 0.694804 | {0.242344, 1.62342} | 60.0896 | ar | 4824. | 17 421.1 | {16 111., 20 167.} | 261.133 |
| 33 | R0 | {1.35084, 6.08841} | {1.99734, 4.42973} | {{0.178098, 0.879678}, {3., 5.97106}} | {91.2318, 40.5089} | inf | 31. | 416.541 | {100.785, 601.644} | 1245.24 | 0.97667 | newly | 44. | 473.77 | {99., 915.} | 981.386 | 0.845345 | Reff | 1.3326 | 0.285262 | {0.0247313, 0.460693} | 78.5936 | ar | 4759. | 16 403.5 | {15 383., 17 605.} | 244.684 |
| 34 | R0 | {1.36912, 6.316} | {1.98987, 4.46947} | {{0.156273, 0.889633}, {3., 5.97033}} | {91.1235, 46.6094} | inf | 47. | 453.828 | {106.627, 949.065} | 867.864 | 0.857705 | newly | 61. | 483.45 | {96., 1067.} | 696.377 | 0.741544 | Reff | 1.33681 | 0.274526 | {0.0237017, 0.46071} | 79.4641 | ar | 4537. | 16 326.8 | {15 434., 18 155.} | 262.26 |
| 35 | R0 | {1.35831, 7.09038} | {2.06219, 3.35237} | {{0.144239, 0.819144}, {3., 5.77737}} | {96.3749, 56.7329} | inf | 102. | 540.217 | {129.572, 1339.59} | 438.924 | 0.608103 | newly | 115. | 635.92 | {101., 1311.} | 465.078 | 0.60629 | Reff | 1.31498 | 0.302874 | {0.0204091, 0.46054} | 77.1356 | ar | 4667. | 16 508.3 | {15 150., 18 418.} | 253.834 |
| 36 | R0 | {1.28315, 5.89938} | {2.39883, 5.33676} | {{1.73157, 1.2752}, {3., 13.1465}} | {89.7703, 63.4176} | inf | 88. | 2571.27 | {502.842, 5772.52} | 2823.1 | 1.29111 | newly | 139. | 2772.21 | {498., 7364.} | 1895.69 | 1.11741 | Reff | 1.25672 | 0.637563 | {0.212228, 1.34359} | 53.7182 | ar | 4460. | 15 715.5 | {7570., 25 660.} | 253.838 |
| 37 | R0 | {1.19303, 2.75113} | {2.29117, 10.8484} | {{1.39426, 4.26793}, {3., 16.9575}} | {101.838, 299.159} | inf | 7. | 2550.24 | {367.768, 5315.79} | 36 332. | 2.34559 | newly | 22. | 2450.88 | {174., 5016.} | 11 043.6 | 1.75928 | Reff | 1.1848 | 1.38935 | {0.291238, 2.21991} | 51.151 | ar | 3142. | 12 832.1 | {6230., 17 641.} | 308.404 |
| 38 | R0 | {1.36033, 7.8088} | {2.12034, 4.42233} | {{1.18849, 2.67904}, {3., 5.13056}} | {67.288, 46.813} | inf | 85. | 1705.04 | {508.416, 5406.95} | 1907.06 | 1.12378 | newly | 88. | 1844.69 | {680., 5469.} | 1998.53 | 1.11942 | Reff | 1.32551 | 0.38593 | {0.0457712, 0.791146} | 74.538 | ar | 4941. | 16 510.9 | {8302., 26 243.} | 235.077 |
| 39 | R0 | {1.27353, 3.70845} | {2.41706, 4.70764} | {{1.67334, 0.838401}, {3., 12.9701}} | {92.6619, 77.8947} | inf | 37. | 2231.48 | {496.088, 5478.8} | 5931.02 | 1.61889 | newly | 69. | 2681.48 | {490., 7261.} | 3786.2 | 1.40185 | Reff | 1.24908 | 0.634098 | {0.207099, 1.34289} | 54.1499 | ar | 3725. | 15 089.2 | {8358., 25 299.} | 306.143 |
| 40 | R0 | {1.19983, 12.1486} | {2.45089, 7.38564} | {{1.16855, 3.3529}, {3., 9.37191}} | {105.78, 39.2058} | inf | 164. | 1056.97 | {566.861, 1975.37} | 552.066 | 0.702729 | newly | 86. | 745.85 | {351., 1813.} | 774.221 | 0.820726 | Reff | 1.07625 | 0.293552 | {0.00489356, 0.626358} | 73.4203 | ar | 3619. | 18 416.3 | {16 721., 23 579.} | 408.879 |
| 41 | R0 | {1.20935, 2.67169} | {2.08422, 9.42496} | {{0.560132, 2.10791}, {3., 13.184}} | {96.2267, 261.175} | inf | 13. | 1705.57 | {290.719, 3666.34} | 13 021.4 | 1.95976 | newly | 45. | 2091.14 | {342., 7049.} | 4550.67 | 1.42969 | Reff | 1.20379 | 1.50817 | {0.246705, 2.9997} | 64.5314 | ar | 3429. | 11 359.4 | {4920., 18 488.} | 231.669 |
| 42 | R0 | {1.23313, 4.74781} | {2.21319, 5.0941} | {{1.48797, 2.43278}, {3., 10.1903}} | {81.4742, 70.1359} | inf | 49. | 409.665 | {175.183, 992.749} | 744.958 | 0.730011 | newly | 79. | 822.63 | {92., 4033.} | 948.671 | 0.644887 | Reff | 1.18196 | 0.673121 | {0.240408, 1.50098} | 57.8297 | ar | 3835. | 18 893.3 | {16 980., 24 981.} | 392.655 |
| 43 | R0 | {1.26105, 2.39713} | {2.20839, 5.42953} | {{0.866663, 2.16073}, {3., 10.06}} | {95.8414, 132.024} | inf | 6. | 2737.78 | {821.408, 5152.57} | 45 529.6 | 2.54409 | newly | 32. | 3708.86 | {1242., 8328.} | 11 491.6 | 1.92248 | Reff | 1.25298 | 1.10849 | {0.340354, 2.17885} | 38.7243 | ar | 3723. | 12 995.6 | {7796., 19 210.} | 250.652 |
| 44 | R0 | {1.36948, 8.88006} | {1.9431, 4.648} | {{0.162708, 0.879754}, {3., 5.99236}} | {90.9611, 55.9223} | inf | 75. | 430.568 | {101.376, 724.746} | 476.993 | 0.643637 | newly | 68. | 449.67 | {84., 1051.} | 565.691 | 0.66553 | Reff | 1.33689 | 0.26441 | {0.0237621, 0.460647} | 80.222 | ar | 4437. | 16 509.9 | {15 454., 18 069.} | 272.586 |
| 45 | R0 | {1.34882, 3.3791} | {1.71244, 7.62459} | {{0.331799, 2.00079}, {3., 16.1394}} | {72.3812, 161.234} | inf | 2. | 706.841 | {119.985, 1365.47} | 35 242.1 | 2.36264 | newly | 4. | 2261.04 | {165., 7191.} | 56 426. | 2.32983 | Reff | 1.34828 | 1.5348 | {0.296968, 2.88194} | 64.3655 | ar | 4422. | 6574.86 | {1550., 10 547.} | 88.4663 |
| 46 | R0 | {1.23774, 4.34917} | {2.33547, 5.7717} | {{0.875885, 2.15942}, {3., 10.0631}} | {101.005, 63.1444} | inf | 31. | 3203.25 | {413.651, 6688.57} | 10 238.3 | 1.86012 | newly | 58. | 4065.77 | {708., 8790.} | 6917.33 | 1.68887 | Reff | 1.22252 | 1.05185 | {0.348777, 1.71976} | 35.3847 | ar | 3685. | 14 051.5 | {8238., 19 851.} | 281.316 |
| 47 | R0 | {1.34327, 4.78811} | {2.13785, 6.04322} | {{0.857964, 2.15987}, {3., 10.0692}} | {82.4185, 52.5061} | inf | 9. | 2903.74 | {772.492, 5093.7} | 32 163.8 | 2.37193 | newly | 13. | 3630.6 | {1180., 6533.} | 27 827.7 | 2.27445 | Reff | 1.33642 | 1.11316 | {0.36612, 2.16724} | 38.7456 | ar | 4517. | 13 167.8 | {8282., 19 646.} | 191.516 |
| 48 | R0 | {1.24366, 4.25814} | {2.22043, 4.68443} | {{1.19087, 2.67893}, {3., 8.95736}} | {84.0166, 36.5752} | inf | 15. | 1555.98 | {572.484, 5199.27} | 10 273.2 | 1.86337 | newly | 19. | 1709.78 | {412., 4981.} | 8898.84 | 1.75465 | Reff | 1.23073 | 0.406497 | {0.0476794, 0.883633} | 70.1201 | ar | 3678. | 15 968.9 | {8054., 22 655.} | 335.723 |
| 49 | R0 | {1.36017, 7.5415} | {2.16845, 9.23872} | {{0.473458, 5.81116}, {3., 14.5129}} | {94.1264, 50.148} | inf | 84. | 485.029 | {36.3572, 612.602} | 494.708 | 0.664319 | newly | 104. | 369.22 | {15., 415.} | 278.038 | 0.502802 | Reff | 1.284 | 0.372574 | {0.0560575, 0.579405} | 70.9834 | ar | 4178. | 17 927.1 | {16 860., 19 126.} | 329.084 |
| 50 | R0 | {1.25333, 6.63578} | {2.23699, 9.71981} | {{0.457095, 5.72904}, {3., 14.6101}} | {107.195, 61.2533} | inf | 101. | 440.765 | {27.4461, 411.88} | 360.628 | 0.582956 | newly | 117. | 336.16 | {11., 345.} | 219.573 | 0.481428 | Reff | 1.14855 | 0.389925 | {0.0908645, 0.67796} | 67.0605 | ar | 3401. | 18 044.8 | {16 989., 19 225.} | 430.573 |
